# Èquipe, community, traditional values, and reproductive rights

**DOI:** 10.3389/fsoc.2022.1043241

**Published:** 2023-01-10

**Authors:** Isabella Corvino, Fabio D'Andrea

**Affiliations:** FISSUF, Università Degli Studi di Perugia, Perugia, Italy

**Keywords:** reproductive health, violence, coercion, discrimination, community

## Abstract

Gender stereotypes still surround women's reproductive health for several reasons. Moreover, in the last 20 years, women's access to essential reproductive healthcare is becoming an issue contributing to inequality and exacerbating different kinds of violence (cultural and structural). The patriarchal system, intimate partner violence, and traditional cultural and conservative values have a huge impact on women's access to contraceptive information, services, and induced abortion. Gender-based human rights violation has a higher impact on those women who are part of a minority or suffer a marginalized social or economic condition. In the present article, the behavior of migrant women's networks is examined as a case study when they come into contact with the health and care sector with the aim to highlight how, in the context of childbirth, they can suffer discriminatory and violent treatment by the community and groups that defend conservative and traditional values due to the exercise of reproductive rights. The investigated context was the Careggi Hospital in Florence, Italy, but in further investigation, the research will take place in different medium-sized cities such as Terni and Perugia. The chosen methodology was that of second-level sources and qualitative interviews with the health personnel who usually deals with these women (two focus groups involving 11 persons of the hospital équipe). This article has two aims: (1) to present the conflict between community behavior and the right to reproductive health and (2) to discuss aspects of IPV as degrading or violent treatment due to the exercise of reproductive rights. The main finding underlined the importance of considering the link between rights, identity, culture, and relationships with the community.

## 1. Introduction

This Brief Research Report article presents original research conducted in 2021 at Careggi Hospital in Florence and some preliminary findings about the nexus between the équipe community, traditional values, and reproductive rights for migrant women. Even in recent decades, it is possible to affirm that it has experienced significant progress in the identification and recognition of rights in sexual and reproductive health from international organizations, which did not result—from a cultural point of view—in a real change in the experienced reality for many women. In recent years Western societies saw administrations being aggressive in seeking to cut funding for sexual and reproductive health and rights while stepping back on laws, policies, and agreements, going far beyond abortion (Girard, 2017; Franklin and Ginsburg, 2019). The focus on the state of physical, mental, emotional, and social wellbeing, and not only the absence of disease, dysfunction, or weakness linked to sexual and reproductive rights, begins to feel quite unrealistic if one thinks that in the last 20 years gender stereotypes surrounding women, direct, cultural and structural violence against women rates have constantly been growing. In a context like the one rapidly outlined, the social attention on these particular women's rights decreased as the pandemic situation forced isolation and reduced time for social relations. It had a great impact on the doctor–patient relationships, even in health structures. Different studies showed that there is a link between social inequality and access to health services and particularly to pre- and post-partum care in the EU (Healthcare Commission Annual Report and Accounts 2008/09, 2009; Appleby et al., 2011) as migrants are more often part of the marginalized or disadvantaged population suffering barriers in accessing obstetric and/or midwifery-led care (Wolff et al., 2008). This case study focused on the behavior of migrant women's community and partners when they come into contact with the health and care sector to understand how difficult this situation can be.

Migrant women's conditions in their destination country are associated with a lack of “communication and connection” to others (Lyberg et al., 2012). Intimate partner violence (IPV) in reproductive health, conceptualized as the negation of women's decision rights in reproductive health, is an issue, mostly if one thinks about the first generation of migrant women who cannot speak the destination country's language, thus experiencing structural and cultural violence. In Tuscany, there are various foreign communities: Sri Lankan, Pakistan, and Indian women generally have problems understanding Italian. When a woman cannot communicate by herself, her only connection is her partner or a member of her community who acts as a filter and a medium between her and the care structure. These circumstances of vulnerability expose women to a high level of stress as they are still subject to the origin country's cultural expectations while needing to adapt to a different environment (Earvolino-Ramirez, 2007; Norris et al., 2009). The necessity to keep alive one's own identity is an issue, but for women coming from patriarchal societies, it can be a dangerous choice. Patriarchal system, cultural and conservative values in some cases are connected to a less informed gender-based human rights environment. Newly arrived people are easily reluctant to talk or even to collaborate in collecting data; cultural mediators or friends are not always available resources. To build up a relation of trust and collaboration, the length of the stay at the care structure or the hospital is as crucial as the relation with the équipe, but unfortunately, migrant women have a low rate of planned and recommended visits. The efforts to prevent the rise of the inequality rate linked to unequal health system access are growing and several good practices are ever more popular. By the way, the culture of origin has a huge impact on women's choices.

**Figure 1:**
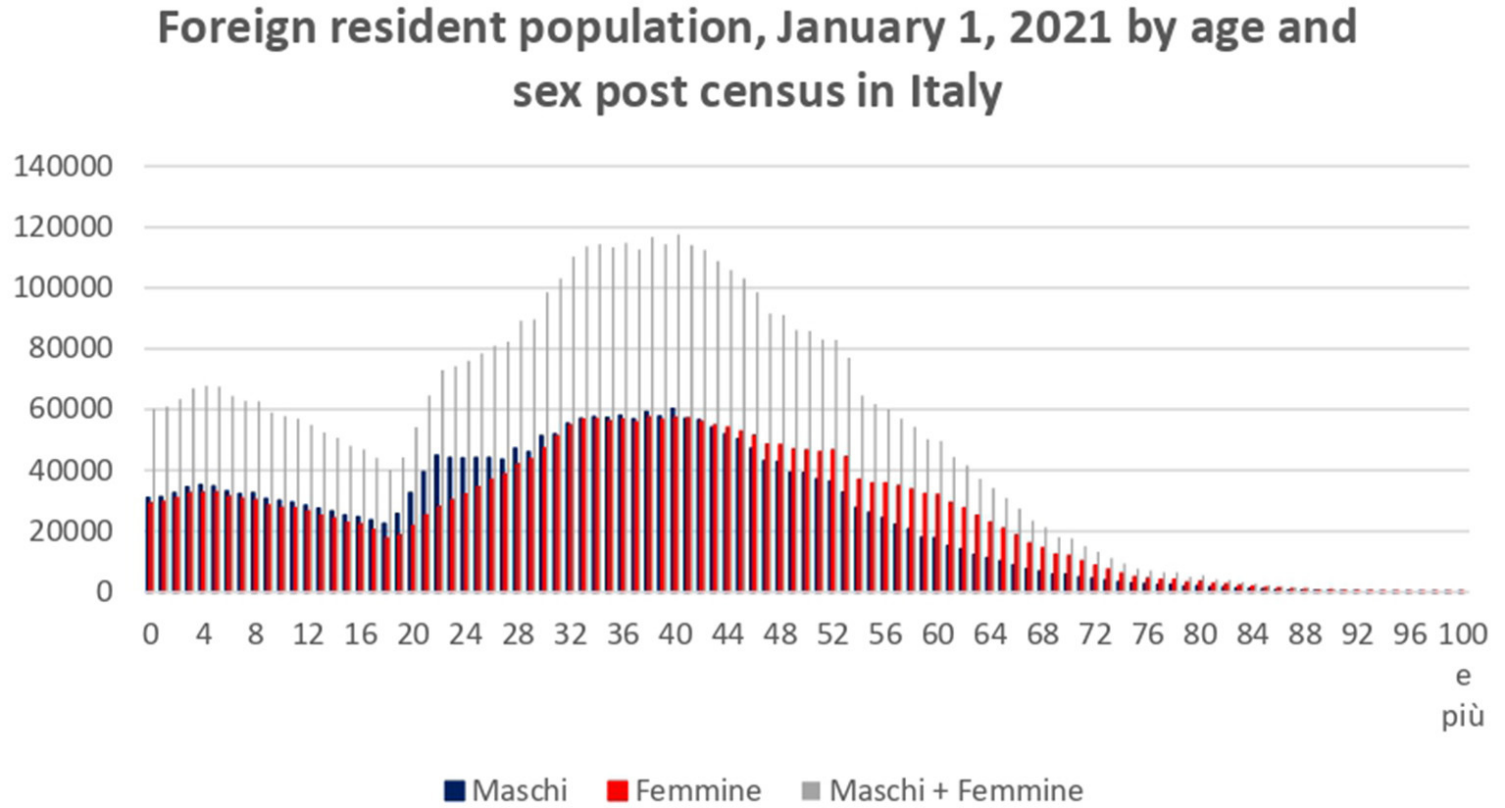
Foreign resident population, Italy 2021. Personal elaboration on Demo Istat data https://demo.istat.it/strasa2021/index.html.

According to the authors, the context of childbirth is a very crucial moment as women's cultural background plays a role in reproductive health (Spadea and Cois, 2004; Mendez et al., 2014). The potential conflict with the origin community and partner can be exacerbated by the need to ensure a better life for the child in contrast to a negative personal experience due to cultural habits. The growing interest in migration phenomena is more and more linked to the ways in which women are implicated in networks and “extended families” (Granovetter, 1973; Wellman and Gulia, 1999; Portes and Rumbaut, 2001). In Italy, the total number of migrants in 2021 is 5.171.894 (ISTAT, 2022, see Figure 1) counting 2.524.644 men and 2.647.250 women, with a higher concentration in the ages 30–45, as shown in the table mentioned later. It is possible to say that migration is a stable reality, with a constant rate as time goes by.

Current flows confirm a growing family dimension (Hartwig et al., 2008); at the beginning, the number of first-generation migrant women who experience pregnancy and childbirth in destination countries is quite high. For the next generation, migrants' behavior tends to be analogous to that of the natives [Certificato di assistenza al parto (CeDAP), 2016]. Foreigners tend to compensate for negative birth rates at the beginning, but in a few years, a reduction in fertility rate tends to occur, an issue that should be investigated. Actually, the usual age of the mother for Italian women is 32.8 years, while it drops to 30.2 years for foreign citizens. Notably, 44.2% have a medium–high level of education, but among foreigners, it prevails a medium–low education (45.9%). From a personal and collective point of view, the interaction with the health sector throughout childbirth is perceived as a significant time (Francisco-Menchavez, 2018). “Migrant women often have unmet social and economic needs during pregnancy, and are more likely to have problems unaddressed by health care systems” (Quintanilha et al., 2016) and even when not against prenatal care, women develop some trust problems with caregivers in part for language barriers and accessing interpreters. Dialog and confrontation are the first steps to building up a trust relation with health personnel; sharing vision, cultures, and data are of absolute importance to elaborate a strategy in the best interests of the mother and the child, in full respect of their rights. Migration data in Italy are non-particularly focused on IPV or reproductive rights; this gap represents a limit to reflection on the issue and to the construction of policies that can improve the situation.

## 2. Method

The field analysis involved privileged observers: 11 health professionals who work or have operated in the birth center of the Careggi hospital in Florence. The study was conducted through the technique of two exploratory focus groups (FGs) taking place in August and September 2021 in remote, as the COVID-19 protocols could not admit interviewers at the hospital. The FG was conducted by the authors and the questions obtained ethical approval from the hospital. The chosen tool could work, even if remote, as an information catalyzer; the interaction between the participants, who already knew each other, was appropriate to bring out plentiful original information, personal stories, and professional experiences. The research aimed at gathering and linking the approaches, evidence, and attitudes of health professionals toward the experience of migrant women's motherhood. All participants (two men and nine women directly involved) had at least 10 years of experience in Italy and abroad (with hundreds of case experiences with migrants indirectly involved). The focus group represents one of the classic tools for qualitative research (Corrao, 2005); this unstructured participatory group interview aims at encouraging people to communicate with other subjects by involving them in the process of reflection and analysis. The results of the FG, collecting data from personal and group experiences, cannot indicate general behaviors, but for this reason, they constitute a privileged place to deepen knowledge of some specific behaviors and experiences. This method is not a representative one from a statistical point of view but is well suited for investigating certain qualitative parts of a topic. The basic idea of this method is that the interaction among interviewees is an important source of information and a privileged point of observation for reflection by participants and interviewers. The interaction also allows for deepening the topics of analysis by proceeding with ever greater levels of detail and allowing more levels of analysis and discussion. The interview went until the saturation point: the section is defined as saturated and representative when, through comparative analysis, all the topics covered by the study are explored and filled, and some information starts to be repeated. One can reasonably say that saturation starts when further questions do not lead to an enrichment of knowledge of the phenomenon but tend to confirm and reaffirm concepts and situations already investigated in previous moments. The talk was also useful for health personnel in elaborating on personal/professional experiences that were, in some cases, very touching. The emotional exposition to the intimate sphere together with the awareness of being the first net dot for problem assessment and first response to dangerous situations can be very demanding but at the same time of incomparable importance to suggest new solutions.

The question framework contains two thematic areas composed as follows:

(a) Structural data of the respondents (sub-area—professional experience/task) and
(b) Migrant women and care systems (sub-area—care relationship/cultural aspects/good practices).

The focus group was oriented to investigate the following:

(a) How do migrant women respond to the care system and in dealing with health personnel?;
(b) Can the different cultural and value systems linked to tradition and implicit knowledge of the care practices create problems?; and
(c) Does the method highlight good practices?

Intimate partner violence was not clearly mentioned as it is a very sensible topic. The dialog during the FG suggested the issue that was treated in the terms chosen by the interviewees.

In our society, the experience of motherhood is usually managed within a medical-healthcare framework and the family and social environment are still crucial. The motherhood experience for migrants emerges in a material and symbolic way as it shows how crossing borders means creating new forms of identity, which are, in any case, hybrid and difficult. When such an important event occurs in a foreign context, in many cases, it changes one's habits and forces to adapt to the new environment. Practical complications are added to psychological vulnerability in some cases, in particular, a condition of double vulnerability: the one experienced by all women in a moment of change like this and the one linked to becoming a mother far from one's own family and culture. The elaborative solitude of migrant women is caused by the loss of external references, while relational exchanges within the family group can be hard. This particular point has been deeply investigated to assess the vulnerability rate of these women in connection with the awareness of their rights. Motherhood generates an experience in which one re-contacts one's own maternal self, in terms of origins and belonging; at the same time, giving birth in another context also means facing the sense of uprooting and the lack of relational affective references to the present. The origin country's culture can be somehow oppressive, but with no connection to the destination country's culture this process, which does not necessarily involve the abandonment of one's knowledge and habits, could be very difficult.

The choice to listen to health personnel instead of migrant women was forced by COVID-19 restrictions and the understanding of the fact that it is still very complicated to invite these women to an open dialog on these topics without putting them in risky situations. Data were processed through transcription, identification of major themes, and the interpretation of experiences and ideas.

## 3. Results

It is not possible to talk about community, traditional values, and reproductive rights, leaving out the relation between the migration phenomenon and the migration network. A great part of the choices made by migrants is elaborated in a dialectic relation with the community of origin in the country of birth and residence. The support migrant can take advantage of is silently linked to a common desire to keep the origin culture alive. After this premise, it is possible to face the issue starting from the migration phenomenon that is a culturally and socially constructed collective project, as it involves migrants, non-migrants, and potential migrants. It is perceived at the same time as an effect of the action of networks and as the result of push and pull factors (social, economic, and political factors/stress). Consequently, being part of the network implies a dynamic position between the micro and the macro level. The social context largely determines individual decision-making processes as it is rooted in a strategy to react to structural determinants (Haug, 2008). Migrating is a decision implying not only the wealth and welfare level of a determined society: The opportunity to count on the care and solidarity network is a very important issue, too (Moro et al., 2012). Migration networks produce different forms of social capital, which ensure the positivity of the migratory experience and can be converted into economic–financial or human capital (Ambrosini, 2020). Family networks play a central role as they represent culture, tradition, material and symbolic support, and crucial points while starting a new family in a vulnerable position. As Cook says, “no society, no religion, no culture and no system of national law has been neutral about issues of human reproduction” (Cook et al., 2003). The reason for this lies in moral, ethical, religious, and identity questions. Sexual and reproductive rights have progressively been recognized in the international arena, but their progress and scope have not always been received without controversy. Migration processes fuel the confrontation between different cultural approaches and law philosophy: in the studied case, origin country's culture and network can be a source of wellbeing and support for the mothers-to-be; but on the other hand, they can be a conservative environment in which these women cannot meet their rights, even if they have the possibility to improve their life quality because coercive and discriminatory practices lead to the instrumentalization of women's bodies. A particularly negative effect can be individuated in the partner's choice to delay women's reproductive rights through degrading or violent treatment with the aim of controlling the woman and her body.

It is quite common that access to public health facilities is not yet well perceived by migrant women because of an “extreme” medicalization of birth; for this reason, migrant women tend not to build a trusting relationship with health personnel. The imagination of birth, the relationship between mother and child, is in their mind “more natural” (citation from the FG); health control and medicines are linked to illness and not to a positive and normal state (Augé and Herzlich, 2013). Different social habits and language barriers can generate misunderstandings; questions about sexual life and birth control could be perceived as inappropriate or absolutely unacceptable, even felt like a form of violence if questions comprehended information about the husbands' behavior. The development of health and sexual rights, as well as medical protocols supporting it, cannot be read in a decontextualized way; they must be analyzed within a broader scene made of social and religious identity. The scarce elaboration on human rights of certain countries is not a mere legislative matter; it is part of cultural and moral norms, and being a migrant can mean bringing part of this baggage to another place, having the feeling of being mistreated by hospital personnel for one's own identity culture can bring the migrant to refuse care and dialog.

Childbirth can be an empowering time in which women, through a dialog with health personnel, other women, and the community network, can estimate the impact and consequences of different approaches and practices, being more aware of rights related to sexual and reproductive health. To take this path, it is essential that their partner does not object or hinder it. Origin community and partners are relevant actors; the challenge that lies ahead in this emancipation process has to do with identity, control, power, and roles and can include violence as “the rupture of the dialogue between self and other” (Cipolla, 1997) among members in case of irreconcilable positions. Childbirth is a peculiar moment in which tradition and identity are not just the celebration of one's roots but the projection of these roots in the future. Assessing the future requires addressing the challenges that lie ahead and that have been present since the initial discussions: identity relations, rights, and self-determination. Women's right to sexual and reproductive health is crucial to their self-government and their right to make significant decisions about their lives and health; unfortunately, policies and protocols are not enough to bring about a decisive cultural change, and more effort is needed to improve women's living conditions.

“Culture can be understood primarily as the way of doing things in people's everyday lives […] it includes knowledge, belief, art, morals, law, literature, lifestyles, ways of living together, value systems, traditions, customs, and any other capabilities and habits acquired by people as members of a society” (Johannes Malesa, 2022, p. 143): Culture is not a given object once and for all, but underestimating the effort needed to overtake such a huge identity question can be naïve. Even patriarchal culture can be perceived as reassuring by women living in a situation of a high level of vulnerability or risk like in a prisoner's syndrome. In patriarchal societies, men's choices are incontrovertible; often, they have a negative influence on women's sexual knowledge, beliefs, and attitudes. Men's position of ownership of women often results in gender inequity, generating violence, marginalization of women while isolating them from other persons with different cultures, and discrimination from others who perceive them as primitive. Cultural negotiation is a crucial step toward a new shared path in the destination country, but linguistic barriers are, unfortunately, the greatest obstacle. Fostering dialog to overcome women's isolation is essential to build up a positive process in the best interest of the mother, the children, and the relation with their origin community.

## 4. Discussion

This study focused on community, traditional values, and reproductive rights, paying special attention to the phenomena that take place within health systems, as they are a valuable space in health promotion and generating culture. Clearly, the duration of the doctor–patient relationship is very important to allow the making of trust and a positive dialog to negotiate information and knowledge. Sociocultural factors shape women's health behavior, and their feelings of not being understood or welcome have a great influence on migrants' health choices. If migrant women feel they are treated differently based on ethnicity or nationality, this can have negative consequences on the delivery of healthcare services (Grove and Zwi, 2006; Willen, 2012). It is not relevant if the feeling is proper or just the consequence of a misinterpretation of reality; for the first generation of migrants, the integration process is yet to be accomplished, and in such a vulnerable state, conformity to traditional values and behaviors can give a sense of protection and correctness. In some cases, coming in touch with the origin country's community abroad can pass through the respect for traditional values as a condition for acceptance (Sayad, 2002). The adoption of the country of destination's cultural practices can be perceived as the craving to abandon one's despised identity and become a member of a different community. It happens that some, particularly, closed communities become almost resistant to the destination country's community; others, on the other hand, can undertake a radical assimilation process (Romania, 2004). A newly immigrant man would be involved in a totally puzzling environment if he did not find firm points of orientation identity with reference to his past life, the stability that can be easily found in their own national group. The analysis of networks' behavior can be very useful in understanding processes of cultural change and assessing the risk connected to the rediscovery or redefinition of identity.

In Europe, health inequalities still remain, more detectable for socio-economic groups and for migrants (Fassin, 2006). These inequalities have been recognized as a priority at the European Council of Lisbon 2000. In Italy, motherhood is usually monitored within a medical-healthcare context, but in health personnel's experience, this attention is not always welcome. Migrants who are not used to this medicalized approach feel uncomfortable with such a great number of clinical exams and control visits: “For many of them, this whole process is exaggerated… there is just a very different attitude, they live in pregnancy in a much more natural and more spontaneous way… Building a dialogue on trust and understanding when imposing protocols and prohibiting traditional practice can be hard… some of them bring at the hospital food and drinks that in our experience can do harm… at the same time it is very common that women who cannot speak Italian and have no network can find at the long-term care unit a support community. Sharing similar experiences and difficulties can push them to feel well disposed to be of some help for other patient. Fairly often some patients played a vital role in acting as cultural mediators, teachers and psychological support persons” (Health Personnel).

As Bonfanti noted, “in our (Western) society, the experience of motherhood is usually managed within a medical framework. […] Migrant motherhood is generally experienced in the perspective of having children who can become Italian citizens, even if starting from culturally different points. These women therefore do not try to recreate the concept of motherhood of their country of origin, they are completely absorbed in the dimension of change. However, the need to maintain the memory of the culture of origin remains fundamental while preserving some of the values that underpin the reproductive experience in it” (Bonfanti, 2012; p. II). The ethos of belonging has its roots in the experience of self, identity, and recognition (Corvino, 2021): every group, as far as identity is concerned, is at the same time an individual and a collective/group construction; this issue, for a negative perception, can produce a painful feeling of disvalue and misrecognition for the minority group. Misrecognition can enhance a condition of double vulnerability (Eriksson and Lindström, 2008), that is to say, the life-changing experience of motherhood as a challenging moment and the becoming mother away from one's own family and culture: “The moment of childbirth is very critical and this does not always help to be open to others, a too intrusive indication can be mistaken as a racist attitude, it is known that a lonely person, perhaps afraid, can easily feel attacked, discriminated. Maybe they are so used to racist attitudes that they have cultivated a vulnerability” (Health Personnel).

It happens to meet women who are poorly supported by their partners as in some cultures childcare is considered a women-only responsibility or a cultural taboo. When language barriers are a real obstacle, these women can face great difficulties: “Sometimes we feel these women avoid questions because these are taboos and they shouldn't talk about it… also because they have no right to a choice. In various cases women, in the hope of giving birth to a son, persisted facing pregnancies even when it was absolutely risky. Sometimes husbands act as translators and filters of medical conversations in order to keep the wife in a controlled cultural contest to limit potential cultural contamination” (Health Personnel).

This preliminary study underlined the importance of considering the link between rights, identity, culture, and relationships with the community. Community and partners can be treasured resources while facing such a challenging experience as giving birth abroad. Culture and dialog among women and health personnel can help them to start a new path and possibly develop a positive relationship with the destination community. Such studies could provide significant insights for better focusing on the integration process and the realization of rights and empowerment while taking into consideration traditional identity and cultural aspects. An effective exploration would need to go beyond the qualitative studies to better assess risks and opportunities in order to elaborate policy and protocol advice.

## Data availability statement

The original contributions presented in the study are included in the article/supplementary material, further inquiries can be directed to the corresponding authors.

## Ethics statement

Ethical review and approval was not required for the study involving human participants in accordance with the local legislation and institutional requirements. Written informed consent to participate in this study was not required from the participants in accordance with the national legislation and the institutional requirements.

## Author contributions

IC: Paragraph 1, 3 and 4. FD'A: Paragraph 2. All authors listed have made a substantial, direct, and intellectual contribution to the work and approved it for publication.
